# Enhanced protection of the renal vascular endothelium improves early outcome in kidney transplantation: Preclinical investigations in pig and mouse

**DOI:** 10.1038/s41598-018-21463-1

**Published:** 2018-03-26

**Authors:** Sofia Nordling, Johan Brännström, Fredrik Carlsson, Bo Lu, Evelyn Salvaris, Alkwin Wanders, Jos Buijs, Sergio Estrada, Vladimir Tolmachev, Peter J. Cowan, Tomas Lorant, Peetra U. Magnusson

**Affiliations:** 1 Department of Immunology, Genetics and Pathology, Uppsala University, Uppsala, Sweden; 2 Immunology Research Centre, St Vincent’s Hospital Melbourne, Victoria, Australia; 3 Department of Medical Biosciences, Umeå University, Umeå, Sweden; 4 Department of Medicinal Chemistry, Uppsala University, Uppsala, Sweden; 5 Immunology Research Centre, St Vincent’s Hospital Melbourne, and Department of Medicine, University of Melbourne, Victoria, Australia; 6 Department of Surgical Sciences, Section of Transplantation Surgery, Uppsala University, Uppsala, Sweden

## Abstract

Ischemia reperfusion injury is one of the major complications responsible for delayed graft function in kidney transplantation. Applications to reduce reperfusion injury are essential due to the widespread use of kidneys from deceased organ donors where the risk for delayed graft function is especially prominent. We have recently shown that coating of inflamed or damaged endothelial cells with a unique heparin conjugate reduces thrombosis and leukocyte recruitment. In this study we evaluated the binding capacity of the heparin conjugate to cultured human endothelial cells, to kidneys from brain-dead porcine donors, and to murine kidneys during static cold storage. The heparin conjugate was able to stably bind cultured endothelial cells with high avidity, and to the renal vasculature of explanted kidneys from pigs and mice. Treatment of murine kidneys prior to transplantation reduced platelet deposition and leukocyte infiltration 24 hours post-transplantation, and significantly improved graft function. The present study thus shows the benefits of enhanced protection of the renal vasculature during cold storage, whereby increasing the antithrombotic and anti-adhesive properties of the vascular endothelium yields improved renal function early after transplantation.

## Introduction

Transplantation of kidneys from brain-dead donors impeded in up to 40% of cases due to delayed graft function (DGF), commonly defined as the need for dialysis during the first week after transplantation^1,2^. Organs from donors after cardiac death are due to warm ischemia exposure even more susceptible to DGF^3^. DGF remains a major hurdle when transplanting kidneys from diseased donors^2^, as it increases the risk of acute rejection^4^ and reduces the overall graft survival^1^.

Ischemia reperfusion injury (IRI), where the pathologic changes occurring during cold ischemia trigger injury upon subsequent reperfusion during transplantation, is a significant contributor to DGF^5^. Development and refinement of organ preservation strategies have reduced the insult to the tubular system that occurs during the hypoxic period of storage prior to implantation^6^. Multiple studies, however, suggest a pivotal role for compromised endothelial cell (EC) function during IRI, pinpointing critical events in the crosstalk between the vascular endothelium and leukocytes^7,8^, platelets^9^, and the coagulation and complement systems^10,11^.

**Figure 1:**
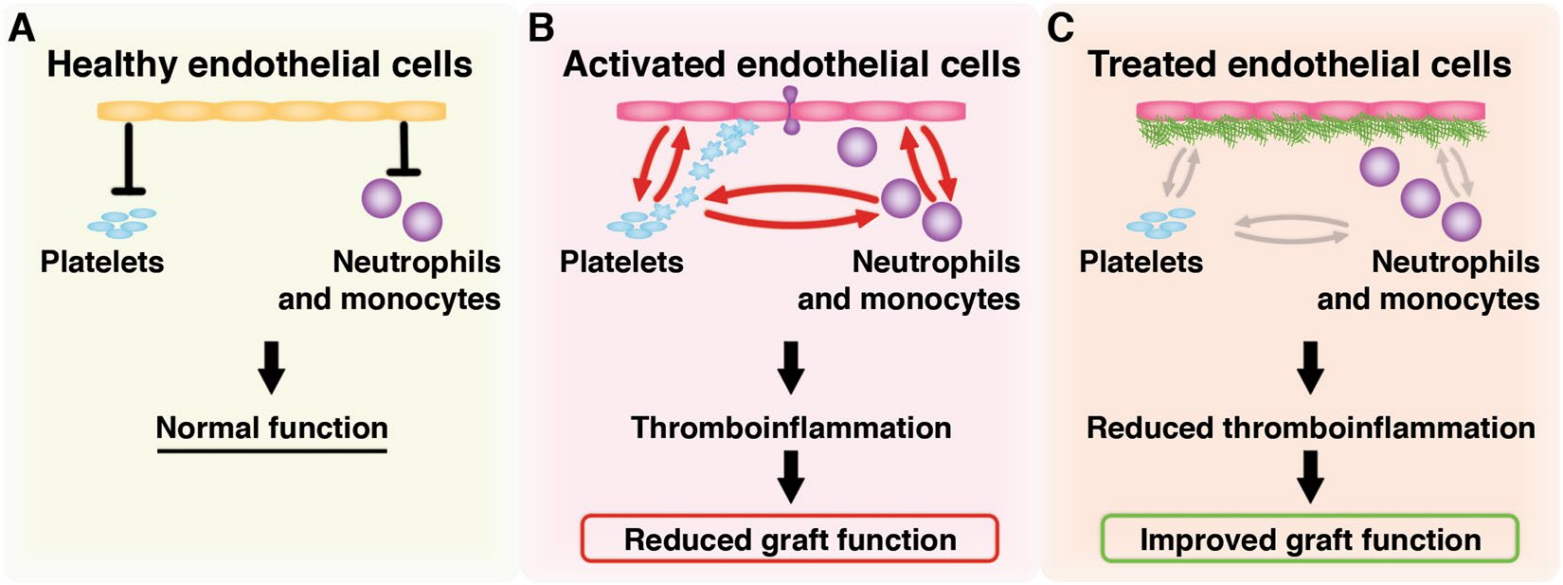
The physiological role of the vascular endothelium and the proposed mechanism of the heparin conjugate, CHC. Healthy EC actively inhibit or regulate the activation and recruitment of platelets and leukocytes (**A**). Activated or dysfunctional EC recruit leukocytes and activated platelets during a process termed thromboinflammation (**B**). Thromboinflammation negatively effects blood flow, and during kidney transplantation it can reduce early graft function. We hypothesized that coating the renal endothelium with the heparin conjugate (green structures in C) prior to transplantation would reduce the impact of the activated endothelium on the blood compartment (**C**). The result would thus be a reduction in thromboinflammation and subsequently, an increase in the early function of the kidney.

Healthy EC line the inner lumen of all blood vessels and operate a vital task in maintaining proper control of hemostasis and inflammation^12^. Hypoxia, in addition to inflammatory cues originating from damaged tubular cells, triggers EC activation, resulting in a more pro-coagulant and pro-inflammatory phenotype of the renal vasculature^13^. Perturbation of normal vascular function occurs immediately upon reperfusion^14^ resulting in platelet and leukocyte recruitment, in a process defined as immunothrombosis or thromboinflammation^15^ (Fig. 1A,B). The consequent disruption of the blood flow, and increased leukocyte infiltration, triggers a further decline in kidney function^16^.

With the aim of reducing inflammatory and thrombotic interactions between the renal vascular endothelium and the blood compartment, we have utilized a heparin-based macromolecule, Corline Heparin Conjugate (CHC). The conjugate consists of >20 unfractionated heparin chains attached to a polyallylamine backbone, resulting in a brush-like structure^17^. The conjugate was originally developed for enhancing the hemocompatibility of artificial surfaces, as the structure of CHC allows binding to a surface while simultaneously ensuring the availability of active antithrombin-binding sites toward blood passing over the surface^18^. We have previously shown that CHC binds cultured EC and reduces recruitment of leukocytes and platelets in addition to reducing the thrombogenicity of hypoxic EC^19^. We have also shown that CHC binds the vascular lining of porcine kidneys when added to the perfusion fluid during hypothermic machine perfusion^20^.

We hypothesized that the conjugate, when added to the preservation solution during cold storage, would form a protective coating on the renal endothelium leading to reduced thromboinflammation and, subsequently, improved graft function early after transplantation (Fig. 1C). In the current study we thus investigated the kinetics of CHC binding to EC, the capacity of CHC to bind to porcine and murine kidney endothelium under static cold storage conditions, and the effect of CHC treatment on the early function of transplanted murine kidneys.

## Results

### CHC binds to endothelial cells with high avidity

**Figure 2:**
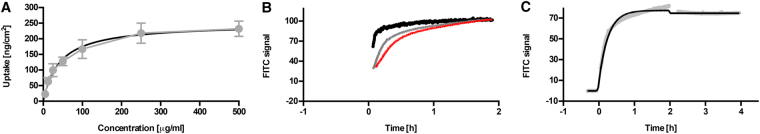
Heparin conjugate binding to human endothelial cells under hypothermic conditions. HDMEC were subjected to a saturation assay with CHC-FITC (**A**). The amounts of CHC bound to the cells (gray line) following incubation with varying concentrations (5–500 μg/mL) of CHC-FITC in UW were fitted with a nonlinear regression model (black line), and the B_max_ was calculated to be 250 ± 20 ng/cm^2^ and the K_D_ to 40 ± 10 μg/mL. Time to reach steady state in the binding (**B**) was 1 h for 500 μg/mL (black line), and 2 h for 100 μg/mL (gray line) and 50 μg/mL (red line) of CHC-FITC. Representative real-time measurement (**C**) of 50 μg/mL CHC-FITC in UW binding to HDMEC, followed by dissociation measured in UW alone. The measured values (gray line) were fitted with a one-to-one binding model (black line), resulting in an apparent K_D_ of 1 μg/mL.

The kinetics of CHC binding to the EC was investigated with a static saturation assay. Human Dermal Microvascular EC (HDMEC) were incubated with increasing concentrations of FITC-labeled CHC in University of Wisconsin (UW) preservation solution under cold storage conditions. The measured amounts of CHC-FITC bound to the HDMEC after 4 h at 4 °C (Fig. 2A; gray line) were fitted with a one-site binding model (Fig. 2A; black line) and the maximal binding capacity (B_max_) of the EC was estimated to be 250 ± 20 ng/cm^2^. The equilibrium dissociation constant (K_D_; the concentration at which half of the binding sites are occupied) corresponded to 40 ± 10 μg/mL.

The kinetics of CHC binding was further evaluated by real-time measurement of the interaction with HDMEC. The cells were again incubated with CHC-FITC in UW at 4 °C as the signal from the accumulating FITC on the cell surface was recorded. The time to reach B_max_ was approximately 2 h for 50 and 100 μg/mL CHC, and 1 h for 500 μg/mL (Fig. 2B). After 2 h, the HDMEC were incubated with UW alone to analyze the dissociation. For each of the tested concentrations, the dissociation rate was very slow (less than 3% per hour) as indicated by the very slow signal decline during the dissociation phase (Fig. 2C; gray line). The resulting association/dissociation curves were fitted with a one-site binding model (Fig. 2C; black line), and the resulting apparent K_D_ amounted to 1 μg/mL. The discrepancy in the estimated K_D_ values between the two binding assays thus suggested that the true binding of CHC to EC is more complex than can be estimated with a one-site-binding model.

### CHC binds to the vascular endothelium in porcine kidneys during cold storage

**Figure 3:**
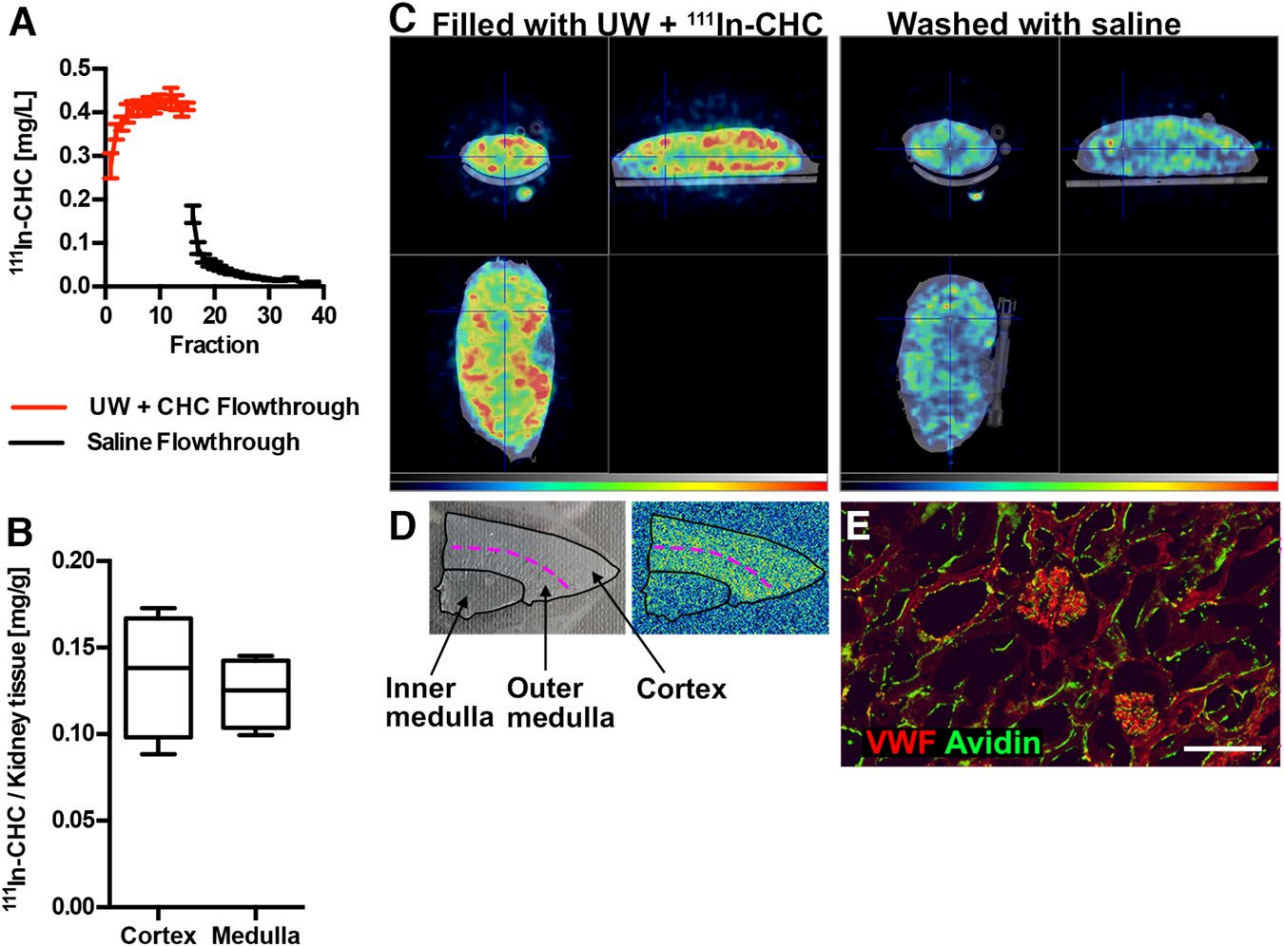
Binding of CHC in kidneys of brain-dead pigs during cold storage. Porcine kidneys were perfused by gravity with UW supplemented with 500 μg/mL CHC, with ^111^In labeled CHC added to trace the conjugate. Fractions of the flow-through from the renal vein were collected during infusion of the conjugate (**A**; red line), and following incubation for 3 h on ice, fractions of the saline used to remove any unbound conjugate were also collected and analyzed in the gamma counter (**A**; black line). The amount of bound CHC was measured in the kidney tissue (**B**) resulting in 0.13 ± 0.02 mg/g binding to the cortex and 0.12 ± 0.01 mg/g binding to the medulla. In total, the kidneys contained 0.13 ± 0.01 mg/g (n = 4). In addition, kidneys (n = 2) were filled with a higher amount of tracer and imaged with Single-photon emission computed tomography (SPECT)/computed tomography (CT) (**C**; left panel, activity gradient in blue–red representing low–high activity). After washing out unbound CHC, the kidneys were imaged again (**C**; right panel) to assess any variation in binding to different regions. The binding was further evaluated by autoradiography. Regions were identified macroscopically on frozen sections (**D**; left panel), and higher amounts were detectable in the cortex and outer medulla compared to the inner medulla (**D**; right panel). Frozen sections were stained with avidin-FITC (green) to detect CHC in the vessels stained for von Willebrand factor (VWF in red: **E**; scale bar = 100 μm).

Next, the feasibility of treating explanted kidneys from brain-dead pigs during cold storage was investigated. The kidneys were infused by gravity through the renal artery with UW containing 500 μg/mL CHC, mixed with a tracer amount of Indium-111 (^111^In) labeled CHC. Fractions of flow-through were collected from the renal vein when CHC was added in order to ascertain that the kidneys were thoroughly perfused with CHC supplemented UW. The fractions were subsequently measured in a gamma counter (Fig. 3A; red line). After incubation for 4 h at 4 °C, the kidneys were flushed with saline, and fractions from the renal vein effluent were again measured in a gamma-counter, to ensure that any unbound CHC was removed from the kidney vasculature (Fig. 3A; black line). The retained radioactivity in the kidney tissue was measured resulting in 10.8 ± 0.87 mg CHC bound per kidney or 0.13 ± 0.01 mg/g when normalized to kidney weight (n = 4). The radioactivity in the medulla was slightly (but not significantly) lower than that present in the cortex (Fig. 3B).

Additional kidneys were treated with the same procedure, but with a higher amount of tracer present, for imaging with SPECT/CT. After filling the kidneys, imaging was performed to estimate the distribution of CHC during treatment (Fig. 3C; left panel). The kidneys were then re-imaged after 4 h of cold storage followed by washing with saline, displaying only the bound conjugate in the kidney (Fig. 3C; right panel). The binding was lower in the innermost part of the medulla as most of the CHC was bound to the cortex and the outer medulla. The difference in the binding pattern was further investigated in frozen sections with autoradiography, confirming that the highest signal originated from the cortex and the outer medulla (Fig. 3D).

In accordance with a previously published protocol^19^, avidin-FITC was utilized in detecting CHC bound to the endothelium. Frozen sections of the porcine kidneys were stained for von Willebrand factor (VWF) to detect the vasculature and with avidin-FITC to assess the localization of CHC. Immunofluorescent staining of the kidney tissue resulted in a distinct FITC signal present in the vasculature of the treated kidneys, particularly in the glomeruli (Fig. 3E).

### CHC binds to the vascular endothelium in murine kidneys during cold storage

**Figure 4:**
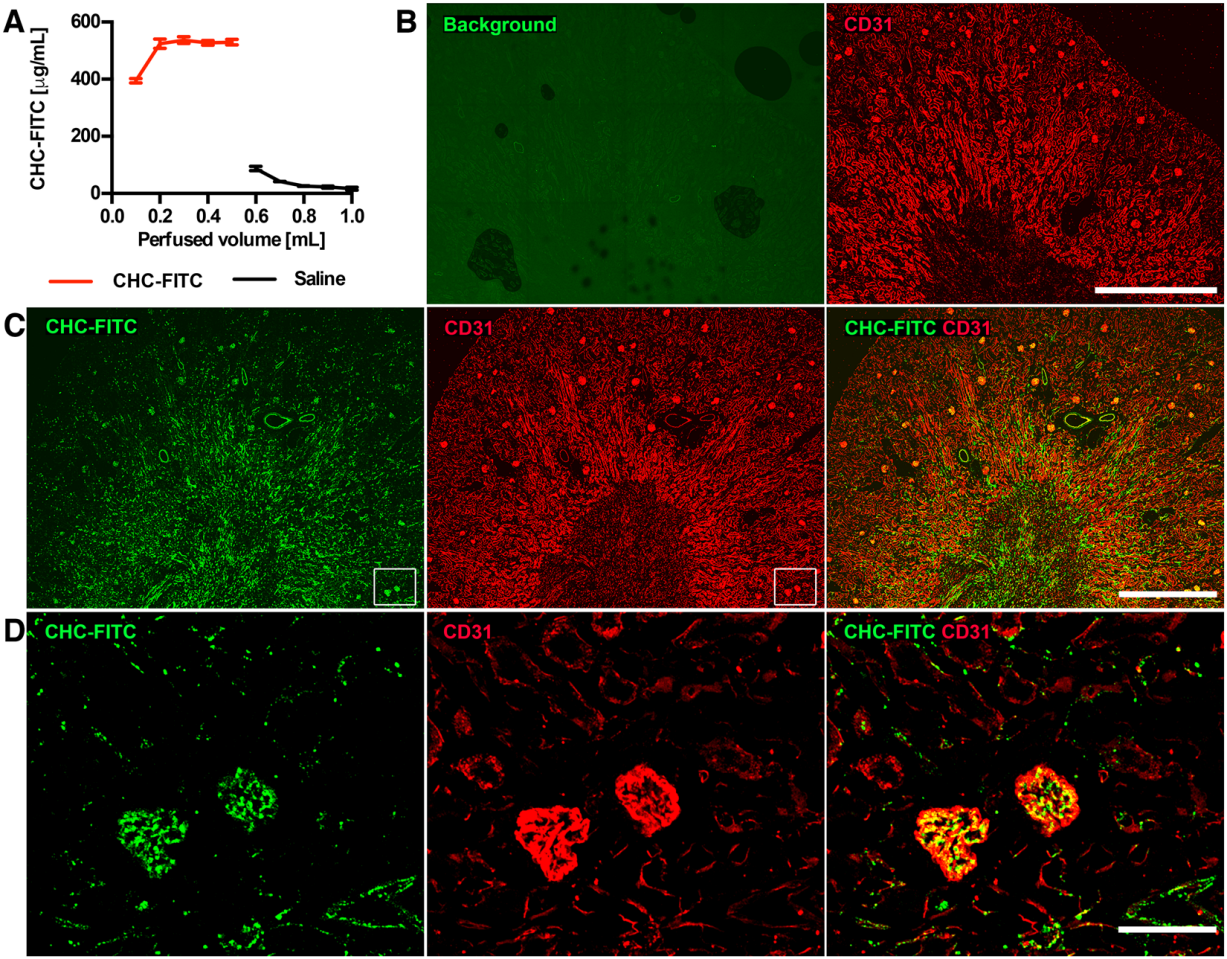
Binding of CHC in mouse kidneys during cold storage. Mouse kidneys were perfused with CHC-FITC, and fractions of the venous flow-through were collected and the fluorescence was measured (**A**; red line). The kidneys were then stored at 4 °C for 5 h, and subsequently washed with saline collected in fractions from the renal vein (**A**; black line). The kidneys were then fixed, sectioned and stained with CD31 (red) in order to detect the vasculature and investigate the distribution of CHC-FITC (green) in mouse kidneys. Kidneys treated with saline alone (**B**) were used as a background control for the FITC signal. Kidneys treated with CHC-FITC (**C**; left- and rightmost panels) had a clear signal in the vicinity of the vasculature (**C**; middle and right panels). Increased magnification showed CHC-FITC in the kidney vasculature (**D**) with some variability in the amount of FITC between different glomeruli. Images are representative of three separate experiments, and scale bars = 1 mm.

Murine kidneys were perfused in a similar fashion as the porcine kidneys, utilizing 500 μg/mL CHC-FITC in saline for visualization of the bound conjugate. Again, fractions of the flow-through from the vein were collected, and the FITC fluorescence was measured to establish sufficient perfusion of the kidneys (Fig. 4A, red line). After subsequent cold storage for 5 h, the kidneys were perfused with saline alone to remove any unbound CHC. The amount of fluorescence in the flow-through was measured again during the washout of unbound CHC (Fig. 4A, black line). Sections of the kidneys were imaged to evaluate the presence of CHC-FITC in the vasculature (stained by CD31; red). Control kidneys had low background fluorescence in green (Fig. 4B) while in CHC-FITC perfused kidneys the majority of the vasculature exhibited binding of CHC-FITC (Fig. 4C,D green), and in contrast to the porcine kidneys, the binding in the inner medulla seemed equal to that of the outer medulla.

### CHC improves early outcome in a murine syngeneic kidney transplantation model

**Figure 5:**
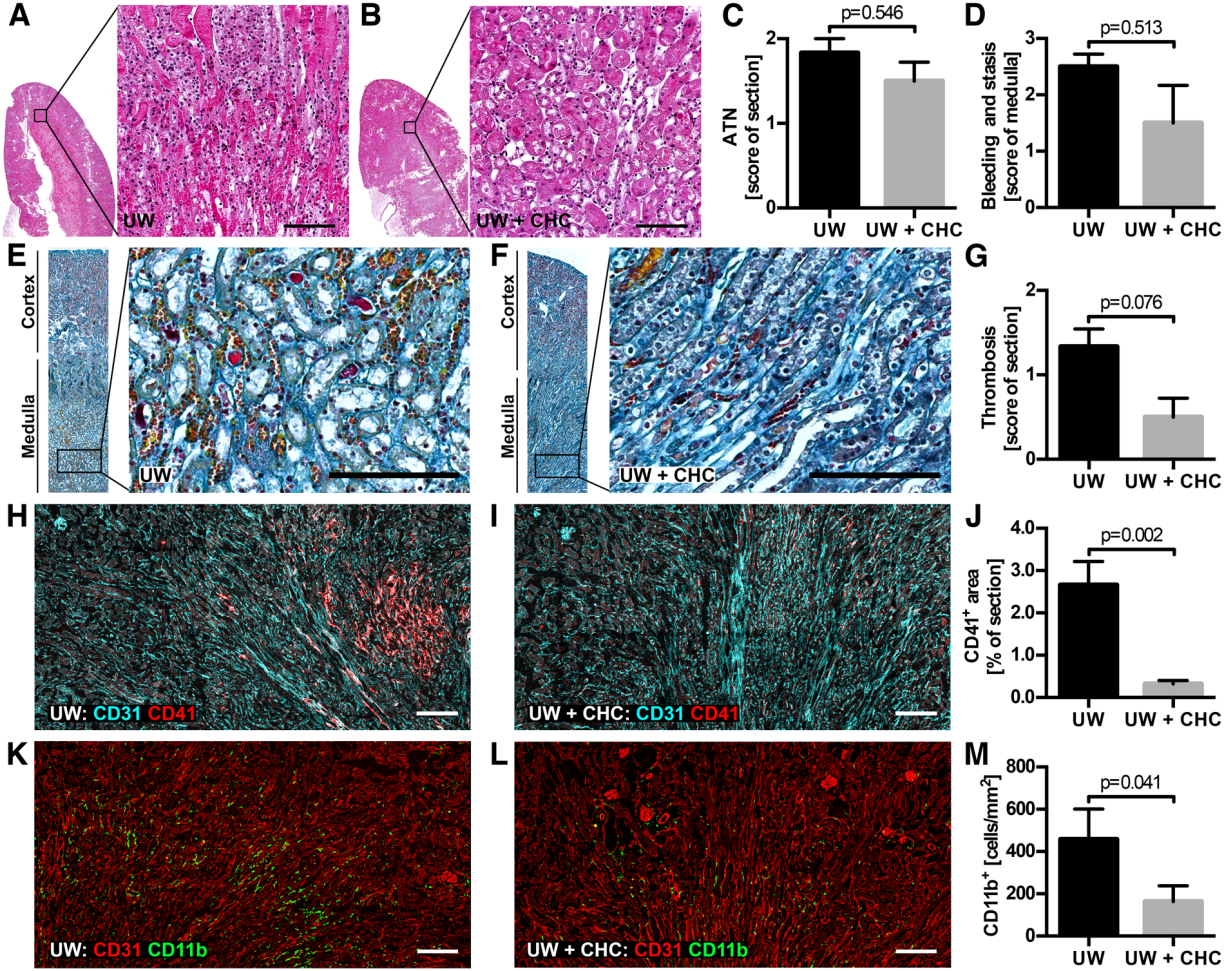
Morphology and cellular infiltration in transplanted mouse kidneys. H&E staining of sections of transplanted kidneys treated with UW alone (**A**) or with CHC in UW (**B**) showed no large differences with regard to histological evaluation of ATN scores (**C**; 1.8 ± 0.2 vs. 1.5 ± 0.2; p = 0.546). There was slightly, but not significantly, less bleeding and stasis of blood in the medulla of CHC-treated animals (**D**; 1.5 ± 0.7) compared to control kidneys (2.5 ± 0.2; p = 0.513). Blinded scoring of fibrin formation in MSB stained sections from control (**E**) and CHC-treated (**F**) kidneys showed a slight reduction in the treated kidneys (**G**; 1.3 ± 0.2 vs. 0.5 ± 0.2; p = 0.076). Frozen sections of the transplanted kidneys were stained for CD41 to investigate the presence of platelets in the control (**H**) and CHC-treated (**I**) kidneys. The total CD41^+^ area in the whole section (half of a longitudinal cross-section) was quantified by image analysis (**J**) resulting in significantly more positivity in the controls (2.66 ± 0.56%) compared to the CHC-treated kidneys (0.32 ± 0.08%; p = 0.002). Whole sections stained for CD11b (**K**,**L**) were analyzed for the total number of infiltrating leukocytes resulting in significantly more CD11b^+^ cells in the controls (457 ± 144 cells/mm^2^) compared to the CHC-treated kidneys (164 ± 75 cells/mm^2^; p = 0.041; **M**). (n = 6 for **A**–**M**, and scale bars = 100 μm for **A**,**B**,**E**,**F** and 200 μm for **H**,**I**,**K**,**L**).

Next, murine kidneys were treated with 500 μg/mL CHC in UW during cold storage and, following rinsing out of unbound CHC, subsequently transplanted into completely nephrectomized recipients, in accordance with a previously published procedure^21,22^. Control animals received kidneys treated only with UW during the 5 h of cold storage. Animals were followed for 24 h after transplantation, during which the mortality was 0%. At 24 h, the kidneys were retrieved and analyzed for the parameters listed in Supplementary Table 1. Blinded scoring of the morphology in hematoxylin and eosin (H&E) stained kidneys (representative images shown in Fig. 5A,B) did not reveal a significant difference with regard to either acute tubular necrosis (ATN; Fig. 5C) or overt bleeding and thrombosis (Fig. 5D). The CHC-treated kidneys did, however, exhibit slightly, but not significantly, fewer fibrin-containing thrombi in Martius, Scarlet and Blue (MSB) stained sections (Fig. 5E–G; scoring = 0.5 ± 0.2) compared to the UW control (scoring = 1.3 ± 0.2; n = 6; p = 0.076).

The presence of platelet driven thrombosis was evident in frozen sections stained for CD41. Control animals had significantly larger CD41^+^ areas (Fig. 5H,J; 2.66 ± 0.56%) in the sections compared to CHC-treated animals (Fig. 5I,J; 0.32 ± 0.08%; n = 6; p = 0.002). Leukocyte infiltration was quantified by staining for CD11b, a surface marker of monocytes, neutrophils and macrophages, all of importance in the inflammatory process. The number of CD11b^+^ cells was higher in control kidneys (Fig. 5K,M; 457 ± 144 cells/mm^2^) compared to CHC-treated kidneys (Fig. 5L,M; 164 ± 75 cells/mm^2^; n = 6; p = 0.041). Both the increased platelet and leukocyte accumulation was mostly located in the medulla. The increased recruitment was accompanied by a slight, but non-significant, change in the expression of inflammatory chemokines and cytokines as observed in RNA isolated from whole kidney tissue (Supplementary Figure 1A–F). Analysis of circulating pro-inflammatory chemokines and cytokines in the serum revealed no significant differences between treated and untreated animals (Supplementary Figure 1G–R).

**Figure 6:**
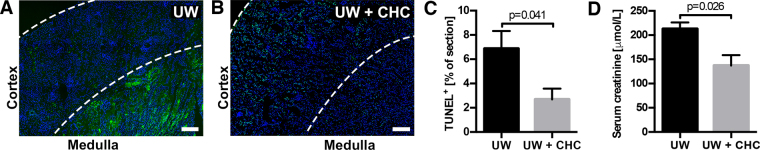
Evaluation of apoptosis/necrosis and kidney function in transplanted mice. Staining for apoptosis/necrosis revealed larger TUNEL^+^ areas (green) in UW treated controls (**A**; DAPI in blue) compared to CHC-treated kidneys (**B**). Image analysis of the TUNEL staining revealed significantly more apoptosis/necrosis in control kidneys (6.87 ± 1.45%) compared to CHC-treated kidneys (**C**, 2.70 ± 0.88%; p = 0.041). Recipients of CHC-treated kidneys had lower levels of serum creatinine at 24 h post transplantation (213 ± 13 μmol/L vs 137 ± 21 μmol/L; p = 0.026), suggesting better kidney function compared to recipients of kidneys treated with UW alone (**D**). (n = 6 for **A**–**D**, and scale bars = 200 μm for (**A**,**B**).

Subtle changes in the presence of apoptosis/necrosis were analyzed with a TUNEL-based staining. Whereas TUNEL^+^ areas were clearly visible in the medullas of the control kidneys (Fig. 6A; 6.87 ± 1.45%), the stained areas were significantly smaller in the CHC-treated kidneys (Fig. 6B,C; 2.70 ± 0.88%; n = 6; p = 0.041). Moreover, serum analysis 24 h after transplantation showed that the recipients of kidneys treated with CHC exhibited significantly lower serum creatinine levels (137 ± 21 μmol/L) compared to the controls (213 ± 13 μmol/L; n = 6; p = 0.026), indicating a higher regain of function in the kidneys treated with CHC (Fig. 6D).

## Discussion

In this study, we investigated a strategy of suppressing the harmful effects of an activated vascular endothelium with the aim of reducing the reperfusion damage of donated kidneys posttransplantation. The treatment consisted of pre-coating the renal vasculature with a unique heparin conjugate CHC prior to implantation. Binding of CHC was stable *in vitro*, thereby facilitating its use during cold storage, whereupon it had an early protective effect in an experimental model of kidney transplantation.

We have previously shown that CHC binds to the apical surface of cultured EC^19^. Here we evaluated the kinetics and properties of CHC binding in detail with both a single-time point saturation binding assay, and an assay that measures binding in real-time. Whereas EC most likely express several binding sites for heparin^23^, the exact number and complexity of these binding sites are still largely unknown, making precise modeling of the binding between CHC and EC unattainable. We therefore applied the simplest model of receptor-ligand binding, which accounts for only one binding site^24^. Nonetheless, we found that the fitted curves corresponded reasonably well to the measured values gathered by either assay. The apparent K_D_ value that was obtained by each of the assays did, however, vary. This suggests that the interaction between CHC and EC is more complex than the utilized model can account for.

As the molecular weight of CHC is unknown, we were unable to calculate the K_D_ in molar units, preventing us from comparing the binding kinetics of CHC with that of other ligands. However, the real-time measurements allowed us to estimate the proportional difference between the association and dissociation rate constants during binding and detaching, respectively. Following binding of CHC to EC, its dissociation was slow with a dissociation rate constant comparable to that of, e.g., therapeutic antibodies that target specific cellular receptors^25,26^. Such a slow dissociation rate suggests a high avidity of the binding, i.e., one CHC molecule binds several binding sites simultaneously and is not released from the cell surface if only one of the bonds is broken. Collectively the results indicated that, in kidney transplantation, CHC would in fact remain bound to the vascular EC upon reperfusion.

We have previously shown that CHC binds to the renal vasculature in porcine kidneys^20^. Here we replicated those initial findings in two separate models of cold storage; the first consisting of kidneys retrieved from brain-dead pigs, and the second of murine kidneys explanted from previously healthy donors. Furthermore, by using ^111^In-labeled CHC in the porcine kidneys, we were able to determine the amount of bound CHC to be approximately 0.13 mg/g kidney tissue - which would ultimately correspond to a dose of 19.5 mg in a human kidney of 150 g. We were also able to visualize the distribution of the bound CHC in the porcine kidneys with SPECT/CT. Given the limitations in spatial resolution of the imaging method, the SPECT/CT images indicated that the kidney vasculature was perfused upon addition of UW with CHC. The SPECT/CT imaging did, however, reveal a heterogeneous distribution of the bound CHC, as surmised by the hot spots (green/yellow/red signal) within the kidney. This heterogeneity was also verified by the autoradiography performed in sectioned biopsies where the majority of signals from the ^111^In-labeled CHC were detected in the cortex and outer medulla. Avidin is a heparin-binding protein and was therefore used to detect CHC^19,27^. Biopsies from the porcine kidneys stained with avidin-FITC showed CHC localized to the blood vessels, and the glomeruli in particular.

In comparison to the porcine kidneys, mouse kidneys perfused with CHC-FITC showed a more equal distribution of CHC between the cortex and medulla. A reasonable explanation for the discrepancy in binding within the murine and porcine kidneys (aside from any difference between the two species) may be the difference in the conditions that the organs were subjected to prior to cold storage. The murine kidneys were explanted from previously healthy donors and the CHC-treatment was immediately applied to the kidneys upon procurement. The porcine kidneys, on the other hand, were subjected to circulatory shock triggered by brain death in the donor. During brain death, released cytokines activate the vascular endothelium, subsequently affecting vasomotor tone, platelet recruitment, and thrombus formation^28,29^. These events may theoretically explain the uneven distribution of perfusion solution to the inner medulla when the kidney is removed from a brain dead donor after cold static storage. On the other hand, CHC is distributed more evenly between the cortex and medulla after cold machine perfusion for 18 hours (unpublished data), which could indicate that the type of storage and the time-point of CHC treatment also affect the distribution pattern in donated human kidneys. Based on these data cold machine perfusion may have some advantages over simple cold storage making it the preferred preservation method in a first-in-man clinical setup.

The effect of CHC on thromboinflammatory events early during kidney transplantation was investigated in a previously utilized mouse model^21,22^. Mouse kidneys that were treated during cold storage were transplanted into nephrectomized, syngeneic recipients for a period of 24 hours. Whereas CHC treatment had no visible effect on kidney histology when compared to controls, there was a slight reduction in the number of fibrinous thrombi. A more notable effect on thromboinflammation was evident in the number of accumulated platelets and leukocytes. CHC-treated kidneys showed a significant reduction in both when compared to controls. Our hypothesis is that CHC upon vascular surface binding provides a substitute to a damaged glycocalyx and thereby prevents platelet and leukocyte interaction with the activated vascular bed and any exposed subendothelial surface. We have earlier shown that binding of the heparin conjugate to both endothelial cells and collagen representing the sub-endothelial layer of the vasculature reduces the recruitment of platelets and leukocytes^19^. Accumulated platelets and leukocytes were mostly located in the highly vascularized outer medulla of untreated kidneys, which is prone to inflammatory damage and reduced blood flow during ischemic events^30^. The significant reduction of apoptotic and necrotic cell death within the medulla in the CHC-treated animals suggested that the renal blood flow was indeed improved compared to the control group. 24 hours post transplantation analyses of either the local gene expression or the systemic presence of circulating pro-inflammatory chemokines and cytokines did not show any significant difference between CHC-treated kidneys compared to control. Despite this discouraging result, some notable differences like a non-significant decrease in IL-1β and a non-significant increase in CXCL10 in animals receiving CHC-treated kidneys were shown. IL-1β is produced by activated macrophages and a lower level of circulating IL-1β may indicate the effect of inhibited leukocyte recruitment. Increased CXCL10 has previously been utilized as a marker for kidney injury^31^, indicating the future need for studies in models that incorporate longer time points post transplantation.

CHC treatment during cold storage had a positive effect on the function of the kidneys, as recipients of CHC treated kidneys had significantly lower serum creatinine levels. The results from the transplantation model thus led us conclude that, given satisfactory delivery of CHC during cold storage, treatment with CHC in UW yields an improved outcome early after transplantation, when compared to UW alone. To date, the optimization of preservation solutions has largely aimed at improving tubular function by reducing cellular metabolism and the subsequent production of reactive oxygen species via supplied antioxidants^6^. However, the damaging effect of IRI still has a major clinical impact in contributing to DGF in approximately 20–40% of patients receiving kidneys from brain-dead donors^5^. Here we conclude that supplementing a standard preservation solution with a treatment that binds and protects the renal vasculature results in improved graft function early after transplantation.

## Methods

### Ethics statement

All work involving animal studies was conducted according to the principles expressed in the Swedish National Board for Laboratory Animals and European Convention for Animal Care. Animal experiments were approved by the Uppsala regional ethics committee in Uppsala Sweden (C325/12, C4/13), and by the Animal Ethics Committee at St. Vincent’s Hospital (Melbourne, Australia) Ltd. (SVHM AEC 028/13r1).

### Labeling with FITC and ^111^Indium

Corline Heparin Conjugate (CHC^TM^, referred to as heparin conjugate or CHC, Corline Biomedical AB, Uppsala, Sweden) was labeled with either fluorescein isothiocyanate (FITC) or Indium-111 (^111^In), see supplemental materials and methods available online for a detailed description.

### Binding assays with cultured endothelial cells

Binding of CHC-FITC to human dermal microvascular EC (HDMEC; #C-12215; PromoCell GmbH, Heidelberg, Germany) was analyzed both with a static binding assay, and in real-time with LigandTracer Green (Ridgeview Instruments AB, Uppsala, Sweden), see supplemental materials and methods available online for detailed descriptions.

### Recovery of kidneys from brain-dead pigs

Healthy male pigs (Swedish landrace 33–36 kg, approximately 12 weeks old) were anesthetized, and brain death was induced according to a previously published protocol^32^, described in more detail in the supplemental materials and methods available online.

### *Ex vivo* perfusion of porcine kidneys

After static cold storage, the kidneys were infused by gravity via the renal artery with 250 mL ice-cold University of Wisconsin solution (UW; ViaSpan; Bristol-Myers Squibb, New York City, NY, USA) containing CHC, supplemented with ^111^In-CHC to a final concentration of 500 μg/mL. The kidneys were incubated with CHC in UW for 4 h on ice, after which 250 mL saline was used to rinse any unbound CHC from the kidneys. Fractions of approximately 10 mL were collected from the renal vein during both infusion of CHC and saline. The fractions were measured in a gamma counter (1480 WIZARD 3”; Wallac, Turku, Finland) to ensure adequate exchange of the liquid inside the kidney. The kidneys were then either dissected into segments of approximately 5 g (consisting of either medulla or cortex) that were then measured in an automated gamma counter, or subjected to Single-photon emission computed tomography (SPECT)/computed tomography (CT) imaging. The total activity of the added ^111^In-CHC was 6 Mega Becquerel (MBq) for measuring binding in a gamma counter, and 30 MBq for imaging with SPECT/CT and autoradiography (see supplemental materials and methods online).

### Procurement of murine donor kidneys

C57BL/6 male mice (aged 12–13 weeks) had access to food and water *ad libitum* before and after the procedures. Donors were anesthetized by intraperitoneal injection of ketamine and xylazine at 16 mg/kg and 8 mg/kg, respectively. The left kidney was used in either *ex vivo* perfusion studies (see supplemental materials) or as the donor kidney for transplantation (see below). Following a midline abdominal incision, the suprarenal and infrarenal aorta and inferior vena cava (IVC) were exposed. Xylocaine (0.5%) was applied around the pedicle to prevent renal vasospasm. A 5–0 thread (Johnson and Johnson Medical, North Ryde, Australia) was placed around the IVC and aorta below the renal pedicle without blocking the blood supply. The left ureter was dissected along its length, free from the kidney hilum to the bladder, and a small elliptical patch of bladder containing the left ureterovesical junction was excised. Donor kidneys were then perfused via the infrarenal aorta with 1 mL cold UW, after which the aorta and IVC below the renal pedicle were tied and the suprarenal aorta and IVC were cut obliquely, approximately 2 mm above the renal pedicle. A standardized time was used for all animals, whereby the whole procurement procedure was 45 min long and included 2 min of warm ischemia.

### Experimental murine syngeneic kidney transplantation model

The transplantation model was adopted from previously published protocols^21,22^. Donor kidneys were excised as described above and perfused with 400 μL UW with or without 500 μg/mL CHC, followed by storage for 5 h at 4 °C. The recipient was anaesthetized and, following a midline laparotomy, for complete nephrectomy the left kidney was removed after ligation of the vessels and cauterization of the ureter and the donor kidney was transplanted orthotopically. The donor aorta and IVC were anastomosed to the recipient’s aorta and IVC end–to-side using continuous 10–0 nylon sutures (Johnson and Johnson Medical). After ensuring correct orientation of the ureter, the dome of the recipient bladder was divided, and bladder-to-bladder anastomosis was performed using interrupted 10–0 sutures. Contralateral nephrectomy was performed following the bladder anastomosis and the abdomen was closed in a single layer with continuous 5–0 nylon or 4–0 silk suture (Johnson and Johnson Medical). Standardized anastomosis times were used for all animals (no more than 30 min for the blood vessel anastomoses, and 20 min for the bladder anastomoses). At 24 h post-transplant, blood was collected for serum creatinine analysis, and the transplanted kidney was explanted and subsequently divided in half and quarter biopsies. Biopsies were either fixed with 10% paraformaldehyde (PFA) for paraffin embedding or 1% PFA for immunofluorescence staining (see supplemental materials and methods).

### Assessment of kidney transplant function

Serum was processed following collection of 400–600 μL blood from the renal vein 24 h post-transplant. Blood was collected in serum separation transport tubes (#365967; Becton Dickinson (BD), North Ryde, Australia), incubated for 30 min at room temperature, and then centrifuged at 6000 × g for 10 min. The serum was collected and stored at −80 °C until analyzed. The serum was thawed at room temperature, and creatinine levels were determined using a kinetic colorimetric assay based on the Jaffé method^33^ and analyzed on a COBAS Integra 400 Plus analyzer (Roche, Castle Hill, Australia) in accordance with the manufacturer’s instructions.

### Statistical analysis

Values were imported into GraphPad Prism and analyzed using the unpaired, nonparametric, and two-tailed Mann–Whitney test with significance defined as p < 0.05. Results were expressed as ± standard error of the mean.

### Disclosures

Dr. Carlsson F. is affiliated researcher at the Department of Immunology, Genetics and Pathology and an employee of Corline Biomedical AB, Uppsala that manufacturers Corline Heparin Conjugate. Dr. Buijs J. is affiliated researcher at the Department of Immunology, Genetics and Pathology and an employee and shareholder of Ridgeview Instruments AB, Uppsala that manufactures and sells LigandTracer.

## Electronic supplementary material

Supplementary information

Supplementary Table 1
