# Supplementary material for: Enhanced protection of the renal vascular endothelium improves early outcome in kidney transplantation: Preclinical investigations in pig and mouse
Source: Sci Rep. 2018 Mar 26;8:5220. doi: 10.1038/s41598-018-21463-1 (PMC5979943; doi:10.1038/s41598-018-21463-1)

Supplementary info

Title: Enhanced protection of the renal vascular endothelium improves early outcome in kidney transplantation: Preclinical investigations in pig and mouse

Authors: Sofia Nordling[1], Johan Brännström[1], Fredrik Carlsson[1], Bo Lu[2], Evelyn Salvaris[2], Alkwin Wanders[3], Jos Buijs[1], Sergio Estrada[4], Vladimir Tolmachev[1], Peter J. Cowan[2], Tomas Lorant[5], Peetra U. Magnusson[1*]

Supplementary methods:

Labeling with FITC and ^{111}Indium

Corline Heparin Conjugate (CHC™, referred to as heparin conjugate or CHC; manufactured and released according to certificate of analysis issued by Corline Biomedical AB, Uppsala, Sweden) was labeled with fluorescein isothiocyanate (FITC) according to the manufacturer's instruction (FluoroTag FITC conjugation kit; Sigma-Aldrich, St Louis, MO, USA). Shortly, 25 µg of lyophilized FITC (isomer I) was reconstituted with 0.1 M carbonate bicarbonate pH 9.0, and vortexed until completely dissolved and diluted 10:1 with the CHC solution (1.2 mg) and incubated for 2 hours at room temperature. The CHC-FITC was purified using zeba spin desalting columns (Thermo Scientific) with PBS pH 7.4. For labelling with indium-111 (^{111}In, Mallinckrodt Pharmaceuticals, Solna, Sweden), CHC was first conjugated with the radiometal chelator cyclohexyl-diethylenetriaminepentaacetic acid (CHX-A"-DTPA) (Macrocyclics, Dallas, TX, USA), enabling subsequent binding of the radioisotope. Before addition of ^{111}In, the CHC-CHX-A"-DTPA was run through by size exclusion chromatography on NAP-5 columns (Amersham Biosciences, Uppsala, Sweden) with metal free 0.2 M ammonium acetate buffer pH 5.5 to remove any unbound chelator. The CHC-CHX-A"-DTPA was incubated with ^{111}In during 1 hour and thereafter the ^{111}In-CHC-CHX-A"-DTPA was separated from nonreacted ^{111}In by using NAP-5 columns.

Static saturation binding assay to cultured endothelial cells

Human dermal microvascular endothelial cells (HDMEC #C-12215; PromoCell GmbH, Heidelberg, Germany) were cultured in complete endothelial cell growth medium microvascular (ECGM MV #C-22020; PromoCell) and used until the 6th passage. For quantification of the uptake of CHC on HDMEC, 50,000 cells were seeded in 12-well plates. Triplicate wells were treated with increasing concentrations of CHC-FITC diluted in University of Wisconsin preservation solution (UW; ViaSpan; Bristol-Myers Squibb, New York City, NY, USA) for 4 h at 4 °C. After treatment, the cells were washed twice with phosphate buffered saline (PBS) and subsequently lysed with radio-immunoprecipitation assay (RIPA) buffer (Thermo Scientific, Rockford, IL, USA). Lysates were transferred to a 96-well plate for quantification of the FITC content with a Victor^2 fluorescent reader (PerkinElmer, Waltham, MA, USA). The background, consisting of cells treated with UW alone, was subtracted from all samples, which were then compared to a standard curve of known CHC-FITC concentrations. The experiment was repeated either three or four times for each CHC concentration, and results were imported into GraphPad Prism (GraphPad Software, La Jolla, CA, USA) and fitted with a function of the specific binding by nonlinear regression, assuming only one binding site on the endothelial cells, as presented in equation 1:

$$\boldsymbol{L} + \boldsymbol{R} \underset{\boldsymbol{k_d}}{\overset{\boldsymbol{k_a}}{\rightleftharpoons}} \boldsymbol{LR}\ , \qquad (1)$$

where L is unbound CHC, R is a binding site on the endothelial cells, LR is the complex formed between CHC and the binding site, k_a is the association rate constant, and k_d is the dissociation rate constant.

Real-time binding measurement with LigandTracer Green

In total, 300,000 HDMEC in 4 mL ECGM MV were seeded in a 10-cm cell culture dish placed in a tilted position to allow the cells to adhere only to a section of the dish. After 3–4 h, unattached cells were removed, and fresh medium was added as the dish was returned to a horizontal position and incubated overnight. Binding was monitored over time at 4 °C with LigandTracer Green (Ridgeview Instruments AB, Uppsala, Sweden) with CHC-FITC binding being proportional to the signal derived from the cell-containing dish position minus the signal derived from the noncell-containing dish position. After establishing a baseline with 3 mL UW in the dish, CHC-FITC was added to a final concentration of 50, 100, or 500 μg/mL. Association was monitored for approximately 2 h, after which the dissociation was measured by replacing the CHC-FITC-containing solution with UW alone. The binding and dissociation of CHC-FITC for each concentration was measured two or three times, and results were analyzed in TraceDrawer (Ridgeview Instruments AB), again assuming only one binding site by using a one-to-one binding model according to the relationship presented in equation 1.

Recovery of kidneys from brain-dead pigs

Healthy male pigs (Swedish landrace 33–36 kg, approximately 12 weeks old) were sedated by an intramuscular injection of xylazine (2.2 mg/kg; Rompun; Bayer, Leverkusen, Germany) and zolazepam (6 mg/kg; Zoletil; Virbac, Carros, France), followed by an intravenous injection of ketamine (25 mg; Ket-amino; Intervet AB, Boxmeer, the Netherlands) and morphine (20 mg; Meda, Solna, Sweden). General anesthesia was maintained during subsequent procedures by infusion of pentobarbital (8 mg/kg/h; Apoteket, Uppsala, Sweden), morphine (0.5 mg/kg/h), and pancuronium bromide (0.25 mg/kg/h; Pavulon, Organon, Oss, the Netherlands) together with

Ringer acetate solution (10 mL/kg/h; Fresenius Kabi, Stockholm, Sweden) through the jugular vein. The animals were ventilated mechanically, and cardiac activity was observed by electrocardiography. Both peripheral oxygen and carbon dioxide pressures in addition to the mean arterial pressure were continuously monitored via the subclavian artery.

The intracranial pressure was increased in a stepwise manner by inserting a Foley catheter in the intracranial space and subsequently inflating the balloon with saline (3 mL/h) while observing the change in pressure with a multiparameter Neurovent-Pressure Temperature Oxygen (PTO) probe (Raumedic, Münchberg, Germany). Animals were considered brain dead when the intracranial pressure surpassed the mean arterial pressure. After 2 h laparotomy was performed and both kidneys were excised and perfused with UW *ex vivo*. The renal artery and vein were then fitted with T-adaptors with Luer connections before the kidneys were stored under static conditions in containers with cold UW for 20–24 h.

SPECT/CT imaging

Single-photon emission computed tomography (SPECT)/computed tomography (CT) imaging was performed using the Triumph Trimodality system (TriFoil Imaging Inc., Northridge, CA, USA), an integrated small-animal positron emission tomography (PET)/SPECT/CT scanner. The settings for the CT scan were 50 kV, 2 frames, and 512 projections. SPECT acquisition was performed using 5-pinhole 75A10 collimators with a dual-head, solid-state, cadmium zinc telluride detector. The acquisition parameters for SPECT were 64 projections × 15 s. SPECT raw data were reconstructed with FLEX SPECT software (TriFoil Imaging), which uses an ordered-subset expectation maximization iterative reconstruction algorithm. CT raw files were

reconstructed by filtered backprojection. SPECT and CT data were fused and analyzed in PMOD 3.5 (PMOD Technologies Ltd., Zurich, Switzerland).

***Ex vivo* autoradiography**

Biopsies were obtained from the kidneys imaged with SPECT/CT. The biopsies were immediately covered with CO_2 powder and stored at –80 °C until use. For *ex vivo* autoradiography, 20-μm thick sections were generated with a microtome, placed onto glass slides, dried, and exposed for 7 days (2.5 half-lives of ^{111}In) to storage phosphor screens (Super Resolution; Perkin Elmer, Downers Grove, IL, USA). The plates were scanned in a Cyclone Plus imager (Perkin Elmer) with a resolution of 600 dpi.

Immunofluorescence staining of porcine biopsies

Fixed porcine kidney biopsies were washed thoroughly in PBS and then immersed in 30% sucrose in PBS at 4 °C overnight. Thereafter, biopsies were frozen and stored at –80 °C until sectioning, when 7-µm sections were prepared and stored at –20 °C until staining. Sections were permeabilized in PBS with 0.05% Tween for 10 min following blocking with 1% bovine serum albumin (BSA) for 30 min. Sections were stained for 45 min at room temperature with rabbit anti-human von Willebrand Factor (VWF, 1:500, #A0082; Dako, Glostrup, Denmark) following secondary goat anti-rabbit 568 (1:500, #A11011; Life Technologies, Carlsbad, CA, USA) diluted in blocking buffer. Sections were thereafter incubated for 30 min at room temperature with avidin-FITC (1:1000; Pierce Biotechnology, Rockford, IL, USA).

***Ex vivo* perfusion of murine kidneys**

Donor kidneys were carefully perfused with 500 µL cold saline with or without 500 µg/mL CHC-FITC and fractions of approximately 100 µL of the flow-through were

collected from the renal vein. Following storage at 4 °C for 5 h, the kidneys were perfused again with 500 μL cold saline while fractions of the flow-through were collected. The kidneys were fixed in 1% paraformaldehyde (PFA) for 24 h and prepared for immunofluorescence staining. The FITC signal in the venous flow-through was compared against a standard curve of known concentrations of CHC-FITC with a FLUOstar Omega microplate reader (BMG LabTech, Mornington, Australia).

Histological scoring of transplanted murine kidneys

Longitudinal 4-μm paraffin tissue sections from the explanted kidneys were stained with hematoxylin and eosin (H&E) according to routine procedures by SciLifeLab Tissue Profiling (Uppsala, Sweden), and with Martius, Scarlet and Blue (MSB; Atom Scientific, Hyde, UK) according to the manufacturer's instructions. A pathologist, with expertise in transplant pathology, carried out blinded scoring of the (H&E) staining as follows: The area containing acute tubular necrosis (ATN) was estimated with regard to the whole section. Scores were issued according to the presence of ATN as follows: no ATN changes at all (score: 0), ATN changes seen between 0-33% of the whole renal section (score: I), between 34-67% (score: II), or more than 67% of the whole renal section (score: III). The presence of acute blood stasis and bleeding was most prominent in the inner zone of the medulla and, hence, these changes were scored in a similarly way but with regard to the area only (score: 0-III). Scoring of the early thrombus formation in the MSB stained sections was carried out in a similar, blinded fashion as follows: no thrombus formation (score: 0), early or diffuse thrombi (score: I), and clear thrombosis (score: II).

Immunofluorescence staining of mouse biopsies

Biopsies were fixed for 24 h in 1% PFA at 4 °C, washed thoroughly in PBS, and then immersed in 30% sucrose in PBS. After embedding in optimal cutting temperature (OCT) medium (Tissue-Tech; Sakura Finetek, Zoeterwoude, the Netherlands), the biopsies were frozen, and then 7-µm sections (half of a longitudinal cross-section) were prepared and stored at –20 °C until staining. Sections were blocked and permeabilized in 10% BSA with 0.05% Tween, stained with any of the following: rat anti-mouse CD41-PE (1:100; #558040; BD), rat anti-mouse CD11b (1:500; #MCA711G; AbD Serotech, Oxford, UK), hamster anti-mouse CD31 (1:100; #MA3105; Thermo Scientific), goat anti-rat Alexa Fluor 488 (1:500, #A11006; Life Technologies Carlsbad, CA, USA), goat anti-rat Alexa Fluor 568 (1:500, #A11077; Life Technologies), goat anti-Armenian hamster Alexa Fluor 594 (1:500, #127-585-160; Jackson ImmunoResearch Laboratories, Suffolk, UK), goat anti-Armenian hamster Alexa Fluor 647 (1:500, #127-605-160; Jackson ImmunoResearch Laboratories), or 4',6-diamidino-2-phenylindole (DAPI; 10 µg/ml; Life Technologies). TUNEL staining was carried with the ApopTag fluorescein *in situ* apoptosis detection kit (S7110; EMD Millipore, Billerica, OR, USA) according to the manufacturer's instruction. After staining, all sections were incubated with 0.1% Sudan Black (Sigma-Aldrich) diluted in 70% ethanol for 25 min at room temperature in order to remove the autofluorescence background. Slides were mounted with Fluoromount-G (Southern Biotechnologies, Birmingham, AL, USA) or Prolong Antifade Mountant (Life Technologies).

Imaging and image analysis

Brightfield images were captured with a Leica Biosystems APERIO AT2 scanner (Leica, Bromma, Sweden). Confocal image scans were captured with a Zeiss LSM710 (Carl Zeiss, Jena, Germany). Scans consisted of 3 × 4 tiles with z-stacks obtained with a 20X objective (NA=0.8). Images were analyzed with ImageJ (National Institutes of Health, Bethesda, MD, USA). Briefly, the whole section area was determined by the size of the DAPI stained area. The $CD41^+$ area and the $TUNEL^+$ area were quantified after thresholding, and then divided by the corresponding $DAPI^+$ area. CD11b was quantified as the number of positive particles after thresholding, and normalized according to the corresponding size of the DAPI staining.

QPCR gene expression analysis

First-strand complementary DNA was synthesized using in a two-step reaction. 1.0 μg total RNA was incubated with 0.5 μg Oligo (dT) and 1 μg random primers (Life Technologies, Scoresby, Victoria, Australia) at 70°C for 10 minutes in a 50-μL reaction. After primer binding, 10mM dNTP, 300 U SuperScript III recombinant ribonuclease inhibitor, 60 U RNaseOUT recombinant ribonuclease inhibitor, 0.1 M DTT and 5× first-strand buffer (Life Technologies, Scoresby, Victoria, Australia) was added. Reverse transcription was performed at 42°C for 1 hour and 70°C for 10 minutes.

Real-time polymerase chain reaction was performed in triplicate using the TaqMan Universal PCR Master Mix system and TaqMan® primer-probe sets for IL-1b (Mm00434228_m1), IL-6 (Mm00446190_m1), TNF-a (Mm00443258_m1), CXCL1 (Mm00433859_m1), CXCL2/MIPS (Mm00436450_m1), MCP1/CCL2

(Mm00441242_m1), and endogenous controls GAPDH (Mm99999915_g1) and 18s (Mm03928990_g1) according to the manufacturer's instructions and analyzed using Applied Biosystems 7500 Fast Realtime PCR system (Life Technologies, Scoresby, Victoria, Australia). The relative expression of each gene was calculated as follows: Cycle threshold (Ct) was corrected against the geometric mean of the reference genes glyceraldehyde 3-phosphate dehydrogenase (GAPDH) and ribosomal 18 s (ΔCt). Relative expression is expressed as $2^{-\Delta Ct}$.

Multiplex cytokine analysis

The levels of cytokines and chemokines in mouse serum samples collected 24 hours post-transplantation were measured using Meso Scale Discovery (MSD, Rockville, Maryland, US) V-PLEX mouse cytokine 19-Plex kit (MSD, #K15255D-1). The samples were analyzed according to the manufactures instructions. In short, the serum samples were pre-diluted 1:4 or 1:10 in Eppendorf low-bind tubes. After activation of the plates, the samples were added to the plates and incubated overnight at 4°C on a shaking plate set to 750 rpm. After washing the secondary antibodies were added and incubated for two hours at RT and then washed to be ready for the reading buffer. The plates were read and analyzed on MSD Sector Imager 2400 within 5 minutes after the addition of the reading buffer. Cytokines and chemokines undetectable above the lowest detection range in the standard curve were excluded in the analysis (IFN-γ, IL-2, IL-4, IL12p70, IL-27 and IL-9). The data was the further analyzed using GraphPad Prism.

Supplementary table 1. Results of analyzed parameters of murine kidney biopsies.

The table shows the analyzed parameters for each of the mice in the study.

Supplementary figure 1. Chemokine and cytokine expression and serum concentrations. The local gene expression within the transplanted kidneys was analyzed by QPCR (**A-F**): IL1B (**A**; 0.143 ± 0.013 vs. 0.105 ± 0.031), TNFA (**B**; 0.022 ± 0.003 vs. 0.026 ± 0.008), IL6 (**C**; 0.379 ± 0.093 vs. 0.205 ± 0.069), CXCL1 (**D**; 1.104 ± 0.169 vs. 1.054 ± 0.150), CXCL2 (**E**; 0.774 ± 0.181 vs. 0.504 ± 0.119), CCL2 (**F**; 0.261 ± 0.065 vs. 0.210 ± 0.023). The systemic concentration (pg/mL) of chemokines and cytokines was analyzed in serum collected 24 hours port transplantation (**G-R**): IL-1β (**G**; 4.29 ± 1.65 vs. 2.17 ± 0.62), TNF-α (**H**; 12.01 ± 1.02 vs. 12.94 ± 0.64), IL-6 (**I**; 728.5 ± 326.1 vs. 705.5 ± 171.0), CXCL1 (**J**; 74.82 ± 22.17 vs. 80.22 ± 12.92), CXCL2 (**K**; 103.60 ± 18.41 vs. 96.06 ± 12.78), CCL2 (**L**; 121.60 ± 14.14 vs. 159.00 ± 62.16), CCL3 (**M**; 5.74 ± 1.65 vs. 5.52 ± 0.93), CXCL10 (**N**; 96.11 ± 12.51 vs. 137.20 ± 12.79), IL-5 (**O**; 10.82 ± 3.67 vs. 14.03 ± 4.40), IL-10 (**P**; 54.09 ± 4.24 vs. 65.31 ± 10.75), IL-15 (**Q**; 39.85 ± 5.96 vs. 36.35 ± 5.47), IL-17 (**R**; 3.02 ± 0.35 vs. 2.56 ± 0.17). (n = 6 for **A-R**.)

Supplementary Table 1:

	Mouse #	**ATN** [score of H&E]	**Bleeding and Stasis** [score of H&E]	**Fibrin formation** [score of MSB]	**CD41** [% section]	**CD11b** [cells/mm^2]	**TUNEL** [% section]	**sCr** [µmol/L]
**UW**	1	2	3	1	1,5	438,6	11,0	226
	2	1	2	1	2,3	272,1	7,1	261
	3	2	3	2	2,8	414,9	4,4	232
	4	2	3	1	3,4	199,0	11,2	175
	5	2	2	2	1,1	267,1	4,9	183
	6	2	2	1	4,9	1150,6	2,7	199
**UW + CHC**	7	1	0	0	0,2	62,2	3,6	58
	8	2	3	1	0,6	155,6	0,4	187
	9	1	0	0	0,2	128,4	4,0	105
	10	2	3	1	0,1	21,3	5,7	176
	11	1	0	0	0,3	90,3	2,5	116
	12	2	3	1	0,6	524,5	0,1	182

Supplementary Figure 1:

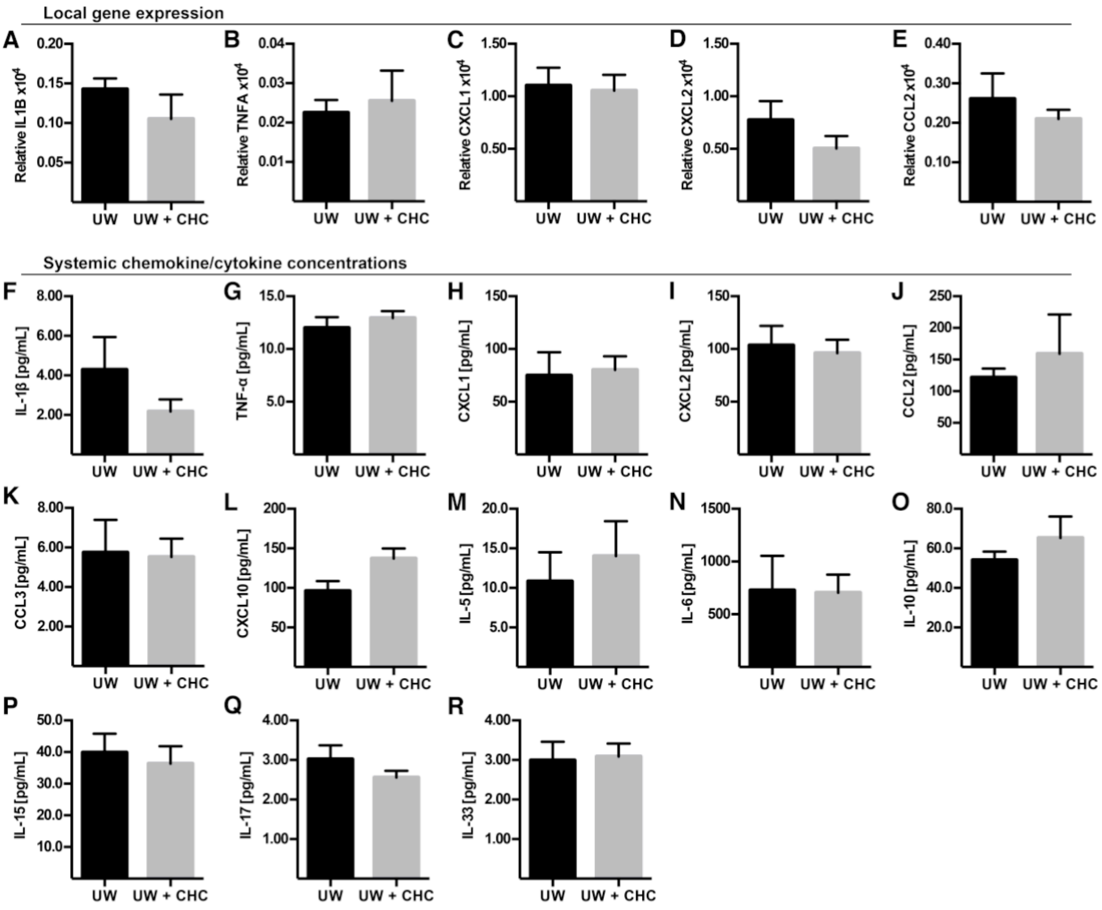

Supplement: Supplementary file 1 — Supplementary information [file 41598_2018_21463_MOESM1_ESM.pdf]
